# HBV-HCC treatment with mRNA electroporated HBV-TCR T cells

**DOI:** 10.1093/immadv/ltab026

**Published:** 2021-12-24

**Authors:** Anthony T Tan, Antonio Bertoletti

**Affiliations:** 1 Emerging Infectious Diseases, Duke-NUS Medical School, Singapore; 2 Singapore Immunology Network, Agency for Science and Technology (A∗STAR), Singapore

**Keywords:** T-cell immunotherapy, hepatocellular carcinoma, hepatitis B, tumour microenvironment

## Abstract

Hepatocellular carcinoma is a significant global health challenge with steadily increasing incidence in the East Asia region. While both Hepatitis C and B virus infections account for the majority of HCC cases, the advent of potent antivirals against HCV infection has biased the aetiology towards chronic HBV infection that at the moment remains without an effective cure. For this reason, HBV-HCC remains a persistent global problem. Treatment options for intermediate to advanced stages of HBV-HCC remain limited, hence novel therapeutic strategies are required to fulfil this medical need. Following the considerable success of adoptive T-cell immunotherapy against B-cell malignancies, it is conceivable to envision whether the same could be achieved against HBV-HCC. In this review, we describe the development of T-cell therapy strategies for HBV-HCC and discuss the safety and the efficacy of the strategies in terms of the direct killing of tumour cells and the other alterations possibly induced by the action of the T cells.

## Graphical Abstract

**Figure:**
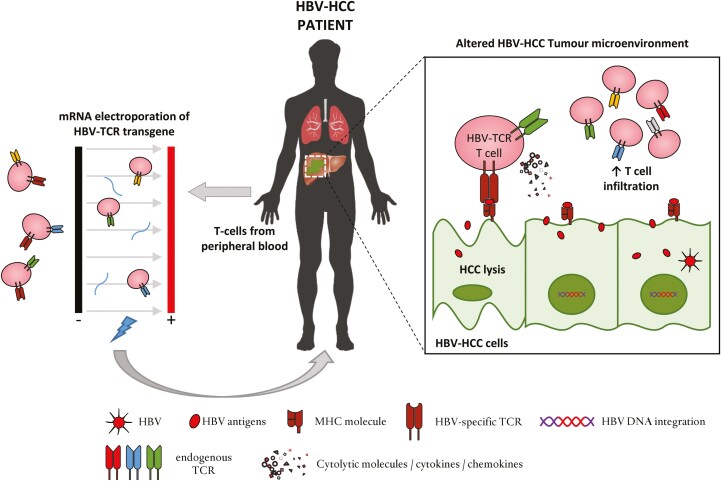

## Introduction

Hepatocellular carcinoma (HCC) is a significant global health challenge with steadily increasing worldwide incidence [1–3] and a disproportionately high number of HCC cases observed in the East Asia region [2, 4]. While the aetiology of HCC is diverse, chronic infection with Hepatitis B (HBV) and Hepatitis C (HCV) viruses accounts for more than 50% of the global cases [2, 4]. With the advent of potent antivirals against HCV infection capable of inducing sustained virological responses and curing a large proportion of treated patients, the incidence of HCC attributed to chronic HCV infection has substantially decreased [5]. In contrast, while existing antivirals (nucleoside/nucleotide analogues) against HBV can potently inhibit viral replication, they do not induce a sustained virological response upon treatment withdrawal and a functional cure (loss of serum HBV surface antigen [HBsAg], of which antigen positivity >6 months is the case definition of chronic HBV infection) is rarely achieved [6, 7]. Other immunomodulatory treatments, like pegylated IFN-α, though less potent in suppressing viral replication, can instead induce a functional cure of chronic HBV infection but only in ~10% of the treated patients [6–8]. As such, without an effective cure for chronic HBV infection, HBV-related HCC (HBV-HCC) remains a persistent global problem.

Treatment of HCC has largely been centred on surgical procedures of chemoembolization, ablation, resection, and transplantation, particularly for the very early (BCLC 0/A) to intermediate stages of HCC (BCLC B), with a desirable expected median overall survival of >2 years post-therapy even in cases of intermediate stage HCC [1]. However, later stages HCC (including HCC recurrence after liver transplantation) have few treatment options, and it is only in recent years where significant advances in the treatment of HCC have been made, particularly in molecular and immune targeted therapies with variable improvements in survival and response rates [9–15]. Considering the still limited efficacy of the available therapies for advanced HCC, the development of novel treatment strategies for HCC is still needed. Of note, the success of adoptive T-cell immunotherapy against B-cell malignancies [16] has raised the question of whether the same could be achieved against HCC. In this review, we describe the development of T-cell therapy strategies for HBV-HCC and discuss the safety and the efficacy of the strategies in terms of the direct killing of tumour cells and the other alterations possibly induced by the action of the T cells.

## Development of T-cell immunotherapy for HBV-HCC

Adoptive T-cell immunotherapy involves the isolation of T cells from the peripheral blood of patients, followed by the gene transfer of receptors conferring a certain specificity to the isolated T cells and the reinfusion of these autologous engineered T cells into the patients. Central to the entire treatment strategy is the appropriate selection of targets to redirect the T cells towards. Ideally, for HCC treatment, the targets should be expressed on HCC cells at sufficient quantities capable of activating the adoptively transferred engineered T cells, with little to no expression on non-tumour cells – to minimize on-target off-tumor effects. Fitting these criteria, a considerable amount of effort has been put into the targeting of tumor-associated antigens (TAA) which are typically fetal or self-antigens (glypican-3, alpha-fetoprotein [AFP] and New York oesophageal squamous cell carcinoma-1 to name a few [17]) that are overexpressed in tumour development and low/absent in normal cells at steady state [18–20]. While this strategy has indeed generated promising initial results in clinics [21–23], off-tumour expression still remains an issue as it is difficult to exclude the low-level expression of TAAs on normal cells or to predict the re-expression of TAAs during dynamic cellular processes like proliferation and inflammation [24, 25]. Hence there is a need to select TAAs with greater specificity for HCC. This was made possible through the discovery of immunogenic HCC-specific neoantigens [26] that arises from mutated self-antigens. Along the course of tumour development, neoantigens can accumulate in the lesion making them highly tumour-specific. However, compared to other tumours, HCC has a low to moderate tumour mutational burden (TMB), which potentially translates to a lower number of possible neoantigens [27]. The tumour specific nature of neoantigens also makes the process of discovering and characterizing them highly complex [26].

An alternative is to draw on the natural etiological link between HCC and chronic HBV infection. During natural chronic HBV infection, the virus frequently integrates its DNA into the human genome [28–30]. HCC cells that developed from HBV infected hepatocytes are therefore marked by expression of whole or partial HBV antigens through a highly tissue-specific process, determined by the hepatotropism of HBV. This makes HBV antigens a more predictable target for T-cell immunotherapy as its expression and consequently, potential on-target off-tumour events, are theoretically confined to the liver compartment with little or no involvement of other organs. From an efficacy standpoint, the stability of HBV integrations during tumor progression [31] compared to the various genetic mutations accumulated during tumour development [32, 33] also favours the targeting of HBV antigens as a more public approach applicable across multiple tumor nodules and patients.

The development of T-cell immunotherapy targeting HBV antigens started with the characterization of chimeric antigen receptor (CAR) specific for the HBV envelope protein [34] followed by other HBV-specific T-cell receptors (TCRs) [35, 36]. To date, many HBV-specific receptors have been well characterized with promising results for the treatment of HBV-HCC [35–40] and some have already entered clinical trials [41, 42]. However, due to the virological background of chronic HBV infection in HBV-HCC patients, the large quantities of circulating HBV proteins in the serum represents a potential risk of neutralizing or activating adoptively transferred HBV-specific CAR T cells. This problem does not occur when HBV-TCRs are used to redirect the specificity of T cells due to the inherent biology of TCR/MHC-epitope engagement. For this reason, the application of the latter approach has an edge for the treatment of HBV-HCC and will be the focus of subsequent discussions.

## Safety of HBV-TCR T-cell immunotherapy in HBV-HCC patients

Careful consideration of safety is necessary when applying HBV-TCR T-cell immunotherapy in HBV-HCC patients, as HBV-specific engineered T cells are unable to discriminate between HBV-infected hepatocytes and actual HBV-HCC cells, giving rise to the possibility of fulminant hepatitis due to the uncontrolled lysis of HBV-infected non-tumor hepatocytes. The potential impact of this issue can be theoretically minimized through the implementation of electroporation to deliver *in vitro* transcribed mRNA (IVT-mRNA) encoding for the HBV-TCRs into T cells. This results in the generation of mRNA HBV-TCR T cells with a limited functional lifespan due to the transient expression of the introduced HBV-TCRs, thereby temporally limiting any treatment-induced inflammatory events [43]. The use of these mRNA electroporated HBV-TCR T cells also allows the HBV-HCC patients to receive multiple infusions of HBV-TCR T cells at escalating doses [42], giving ample time to monitor for unforeseen adverse events occurring at different treatment doses. In addition, safety can be further enhanced when implementing HBV-TCR T-cell therapy in cases of HCC recurrence post-liver transplantation by the careful selection of HBV-TCRs recognizing HBV epitopes restricted by MHC molecules only present in the host and not on the donor liver graft.

Taking into account the safety aspects above, a proof-of-concept trial was conducted in a compassionate setting for the treatment of two chronic HBV patients with HCC metastases post-liver transplantation [42]. In this trial, the appropriate HBV-TCR was selected based on the recipient/donor liver graft MHC mismatch, TCR T cells were engineered through mRNA electroporation and an intra-patient dose-escalation trial design was implemented, all in an effort to maximize safety. It is important to note that for this indication, HBV-TCR T-cell therapy will be implemented in the presence of immunosuppressants that could further subdue the occurrence of adverse events. With these conditions in place, no treatment-related adverse events were observed in both patients after a total of 40 HBV-TCR T-cell infusions. A similarly designed trial (The Third Affiliated Hospital, Sun Yat-sen University, Guangzhou, China) also observed no adverse events in mRNA HBV-TCR T-cell-treated patients with similar indications (personal communication).

The situation is likely to be different when HBV-TCR T-cell therapy is applied in patients with primary HBV-HCC without transplantation, given the absence of immunosuppressants and the shared recognition of HBV epitopes restricted by MHC molecules present on both tumour and non-transformed HBV infected hepatocytes. For this reason, another trial was conducted to evaluate the safety of mRNA HBV-TCR T-cell therapy in patients with diffused non-operable HBV-HCC using a similar dose-escalation infusion protocol (Tan *et al.* 2021 Immunological alterations after immunotherapy with short-lived HBV-TCR T cells associated with long-term treatment response in HBV-HCC. *Hepatology Communications* – accepted and in Press). Out of the eight patients who received multiple infusions of HBV-TCR T cells, one patient developed a localized liver inflammation after receiving a low dose of HBV-TCR T cells (10^5^ TCR+ T cells/kg) with elevations of serum alanine aminotransferase (ALT), total bilirubin, and the development of jaundice for ~25 days. Full normalization of clinical symptoms and liver function was made ~80 days from the onset of inflammation without clinical intervention and subsequent infusions with up to 5 × 10^6^ TCR+ T cells/kg did not induce any liver inflammatory events. Another only showed elevations of ALT without clinical symptoms after receiving a similarly low dose of HBV-TCR T cells. These safety results are very promising as it shows that while HBV-TCR T-cell therapy in primary HBV-HCC patients can sometimes trigger a localized liver inflammation in some patients without systemic involvement, the liver damage was well tolerated and fully reversible despite the advanced pathological liver condition present in the patients.

Hence, with the currently available data obtained through phase 1 clinical trials of mRNA HBV-TCR T-cell immunotherapy in patients with primary HBV-HCC or HBV-HCC recurrence, the risk of systemic adverse events is unlikely and that the induction of liver inflammation can be carefully managed given the transient nature of mRNA HBV-TCR T cells. However, it is also worth noting that the occurrence of liver inflammatory adverse events does not appear to be dose-dependent. This makes the mRNA HBV-TCR dose-escalation approach perfectly suited for the safe implementation of HBV-TCR T-cell therapy in HBV-HCC patients. Clearly, more trials have to be performed to have a definitive safety profile of the treatment for each HBV-HCC indication.

## Efficacy of HBV-TCR T-cell immunotherapy in HBV-HCC patients

Several important observations in terms of anti-tumour efficacy were also made from these phase 1 clinical trials. In the liver transplanted patient with HBV-HCC recurrence [42], infusions of mRNA HBV-TCR T cells caused an observable on-treatment reduction of tumour secreted AFP and off-treatment rebound, consistent with the results from *in vivo* pre-clinical murine xenograft model of HBV-HCC where tumour progression was halted only with continued mRNA HBV-TCR T-cell infusions [43]. Despite this transient reduction of AFP, a durable reduction in total tumour burden was observed through computed tomography (CT) imaging ~2 months after receiving the 7th mRNA HBV-TCR T-cell infusion [42]. This was rather unexpected since the adoptively transferred mRNA HBV-TCR T cells would have long lost the expression of the introduced HBV-TCR and hence can no longer provide any direct anti-tumour effects. CT imaging of the individual tumour lesions in the lung also showed that while some lesions have completely disappeared, others have substantially reduced in size and stabilized without any rebound in tumour size in between T-cell infusions. This was true even when the frequency of T-cell infusions has been reduced (from once every 2 weeks to once a month), or when infusions have completely halted for around a month for monitoring. In addition, analysis of the PBMCs of the patient showed an elevation of the frequency of activated and proliferating T cells ~5 days after infusion. This large magnitude (reaching 10% of circulating CD8 T cells) of activated T cells would suggest that an immunological reaction was induced in this patient by the T-cell therapy, though it was difficult to determine whether this was solely the case or whether it was confounded by the number of activated HBV-TCR T cells infused. Taken together, it was probable that in this patient, the repeated infusions of mRNA HBV-TCR T cells have induced a durable anti-tumour immune response responsible for the continued suppression of tumour growth even when HBV-TCR T cells are absent or no longer functionally active, akin to the widely discussed perpetuation of the cancer immunity cycle.

Bearing in mind the immunosuppression that these patients received, it is conceivable that the efficacy of mRNA HBV-TCR T cells could be more pronounced when it is applied in primary HBV-HCC patients. This was indeed observed in the trial (Tan *et al.* 2021 Immunological alterations after immunotherapy with short-lived HBV-TCR T cells associated with long-term treatment response in HBV-HCC. *Hepatology Communications* – accepted and in press) where four out of eight patients exhibited either a reduction or stabilization of tumour burden after multiple infusions of mRNA HBV-TCR T cells. More importantly, the extent of the anti-tumour efficacy observed appears to be positively associated with the magnitude of immunological alterations induced by the mRNA HBV-TCR T-cell infusion. For example, in the patient who developed the severe liver inflammatory adverse event, a striking activation of peripheral blood CD8 and CD4 T cells was also observed after HBV-TCR T-cell infusion. This same patient also had a dramatic reduction of his liver tumour size after the resolution of the liver inflammation, and tumour burden stabilized for ~7 months even without any infusion of mRNA HBV-TCR T cells. In the other patients with different degrees of observable tumour reduction or stabilization, immunological alterations ranging from liver inflammatory events, peripheral blood T-cell activation, and elevation of serum chemokines secondary to T-cell activation were observed upon treatment.

Hence, while the function of the adoptively transferred HBV-TCR T cells *per se* is clearly important, it does not appear that the functional persistence of the adoptively transferred HBV-TCR T cells is an obligate parameter that determines the anti-tumour efficacy of HBV-TCR T-cell immunotherapy for HBV-HCC. Rather, repeated HBV-TCR T-cell infusions can induce immunological alterations in the patient that seems to be critical. For this reason, it is important to consider that the effects of HBV-TCR T-cell immunotherapy for HBV-HCC can go beyond the initial direct killing of tumour cells, and include alterations of the HCC microenvironment or even the general immune system of the patient.

## Inducing immunological alterations using HBV-TCR T-cell immunotherapy

The microenvironment of HCC is a complex mix of parenchymal, non-parenchymal, stromal, tumour, and both resident and recruited immune cells. These different cellular populations interact dynamically and secrete a myriad of soluble factors, creating a tumour microenvironment (TME) that can profoundly dampen both the adaptive and innate anti-tumour immune response, as evidenced by the accumulation of functionally compromised tumour infiltrating lymphocytes [44] and NK cells [45] that generally correlates with a worse prognosis. While the mechanism of immune suppression is diverse, the HCC TME can be broadly categorized based on the spectrum of immune cell infiltrate. On one hand, you have immune-active HCC that are characterized by ample classical CD4 and CD8 T-cell infiltrates, while on the other, immune-excluded HCC are devoid of effector T-cell infiltrates and instead have an accumulation of cells mediating immune suppression, including T-regulatory cells [46]. Some HCC tumours can also exhibit an immune-exhausted characteristic where T-cell infiltrates are present in the TME but are dysfunctional.

**Figure 1.**
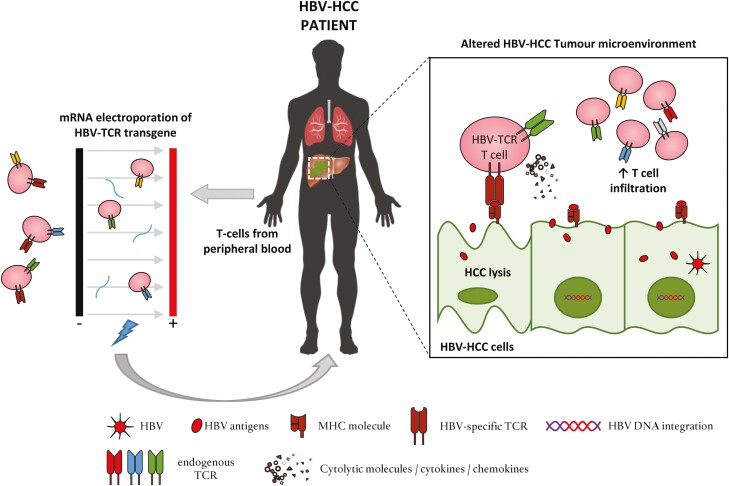
Tumour lysis and alteration of HCC TME by adoptive transfer of mRNA electroporated HBV-TCR T cells. The labile nature of mRNA results in a transient expression of HBV-specific TCR when the T cells are engineered through mRNA electroporation. This limits the functional lifespan of the engineered T cells to ~3–5 days allowing for multiple infusions to be applied in patients. Preliminary evidence suggests that the HBV-TCR T cells not only directly lyse HBV-HCC cells, the resulting secretion of soluble factors might also modify the HCC TME to favour increased immune cell infiltrates that augments the anti-HCC efficacy of the treatment.

The classification of HCC into these broad categories has important clinical implications as a more inflamed tumour status is associated with better response to immune-mediated checkpoint inhibitor therapies in HCC and across various other tumour types [47, 48]. In HCC, an inflammatory TME was also found to associate with slower tumour progression [49], hence it would not be surprising that an ability to convert the TME into one that favours cellular infiltrates could benefit the outcome of immunotherapy approaches including HBV-TCR T-cell therapy. This concept is exemplified in the clinical trials where Atezolizumab (anti-PDL1 blockade) was used alone or in combination with Bevacizumab (anti-VEGF therapy) in the treatment of HCC summarized in a recent review by Sangro *et al*. [15]. The pharmacological blockade of VEGF/VEGFR axis, shown to improve recruitment, trafficking and activation of T-cell response in other solid tumours [50, 51], significantly improved the overall response rate and progressive disease rate compared to anti-PDL1 blockade monotherapy. Given the evidences that mRNA HBV-TCR T-cell immunotherapy was able to induce a durable anti-tumour response despite the transient nature of their functionality, can this approach then be used to modify the TME of HBV-HCC? And are there evidences of such an effect from the limited clinical trials performed? (Fig. 1)

From the various information that have been accumulated about mRNA HBV-TCR T-cell immunotherapy for HBV-HCC, two characteristics of the approach are actually well suited for the modification of HBV-HCC TME. The first is the ability of mRNA HBV-TCR T cells to simultaneously lyse HBV-HCC cells and secrete large quantities of IFN-γ [43]. In HCC patients, lower serum levels of IFN-γ were associated with a more advanced tumour stage, a worse prognosis, and lower recurrence-free survival after curative treatment [52]. Expression levels of IFN-γ receptor on HCC tissues was also negatively correlated with tumour size, serum AFP levels and the occurrence of intrahepatic and extrahepatic metastasis [53]. Hence, it is likely that the intra-tumoral presence of IFN-γ is important for an effective anti-HCC immune response. Since the secretion of IFN-γ by HBV-TCR T cells is induced only after encounter with HBV-HCC cells, this *in situ* delivery of IFN-γ supports the idea that HBV-TCR T cells can possibly modify the HBV-HCC TME. In addition, the direct tumour cytolytic capability of HBV-TCR T cells can further lead to cascades of inflammatory events that stimulate innate and adaptive immune responses, and potentially promote epitope spreading events due to the release of HBV-HCC specific antigens into the now inflamed surrounding, in effect changing the HBV-HCC TME.

The other important characteristic is the repeated replenishment of freshly engineered mRNA HBV-TCR T cells. Since this treatment approach is not dependent on the persistence of the HBV-TCR -cells, it will be less likely for these adoptively transferred T cells to be altered by the disease itself or concomitant medications received by the patient or become functionally exhausted through continuous and repeated activation. While immune checkpoint blockade strategies (alone or in combination with TME modifying anti-VEGF therapy) that attempt to restore the *in vivo* HCC-specific T-cell function can potentially modify the TME as well, the frequent replenishment of T cells recognizing HCC in the patient has a potential advantage in terms of the TME modifying capacity. This is because in this context, immune checkpoint blockade strategies rely on the functional rejuvenation of HCC- or HBV-specific T cells. However, in the background of chronic HBV infection, these HBV-specific T cells are either deleted or functionally exhausted, with a pronounced reduction in their ability to secrete inflammatory cytokines and lyse target cells [54]. This dysfunction extends beyond mechanisms of immune checkpoint receptor engagement to include multiple metabolic alterations that cannot be completely reversed simply by immune checkpoint blockade [55]. Hence, it is likely that the use of immune checkpoint inhibitors can only provide a partial restoration of HBV-HCC specific T cells that might have limited TME modifying capability.

Unfortunately, without studies designed specifically to evaluate the inflammatory status of HBV-HCC before and after T-cell immunotherapy, it is difficult to determine whether adoptively transferred mRNA HBV-TCR T cells can indeed modify the HBV-HCC TME. However, some indirect evidences from initial clinical trials do point to this possibility. As stated in the previous section, various immunological alterations were observed ~3–5 days after mRNA HBV-TCR T-cell infusion in primary HBV-HCC patients who subsequently had a reduction in tumour burden or a stabilization of the disease. While this does not directly reflect an alteration of the TME, it does draw an association between modifications of the immune system of the patient and an observable change in the tumour. Furthermore, in the patients who developed liver inflammation after receiving a low dose (10^5^ TCR+ T cells/kg) of HBV-TCR T cells, it is unlikely that the amount of cytolysis mediated by the small number of HBV-TCR T cells could sustain the abnormal serum ALT and total bilirubin levels observed for over 2 weeks (patient with mild liver inflammation) or ~80 days in the patient with a severe liver inflammation. This is especially so given the transient function of mRNA HBV-TCR T cells. Surprisingly, subsequent infusions of mRNA HBV-TCR T cells no longer induced any liver inflammation in both patients even when infusion doses were increased up to 50-fold (1–5 × 10^6^ TCR+ T cells/kg). Clearly, the lack of any induction of liver inflammation cannot be explained by the absence of HBV-TCR T-cell targets as the hepatic tumours were not completely eradicated, and HBV envelope protein remains detectable in the serum indicating the continued presence of HBV infection. Hence, it is likely that the initial prolonged liver inflammation and subsequent absence of liver inflammatory events were a consequence of TME changes triggered by the activity of the small numbers of HBV-TCR T cells infused, supporting the ability of mRNA HBV-TCR T-cell therapy to induce TME modifications.

## Conclusion

Immunotherapy of HBV-HCC using T cells engineered to transiently recognize HBV epitopes is a treatment strategy with a good safety profile and promising efficacy in patients with either primary HBV-HCC or HBV-HCC recurrence after liver transplantation. While it is still necessary to evaluate both safety and efficacy in a larger number of HBV-HCC patients, observations from initial clinical trials suggest that, in addition to the lysis of HBV-HCC tumour cells, the approach could also have the ability to modify the HBV-HCC TME. This added ability could potentially work in concert with existing first-line HCC treatments using anti-VEGF and immune checkpoint blockade strategies and even new strategies to augment the anti-HCC efficacy. For example, improvements in HBV-TCR T-cell therapy were demonstrated recently in a proof-of-concept study where HBV-TCR T cells were engineered to resist the effects of immunosuppressants [56]. Here, electroporation of mRNAs coding for HBV-specific alpha/beta TCR and mutant versions of immunosuppressive drug targets were able to simultaneously and reversibly modify T-cell specificity and improve effector functions in the presence of immunosuppressants. This would potentially improve the efficacy of treatment for patients with HBV-HCC recurrence after liver transplants who are under immunosuppressive treatments. In addition, the expression and/or function of endogenous proteins can also be transiently altered through the use of splice-switching antisense nucleotides whereby TCR-T cells with reduced cytolysis [57] or capable of secreting soluble forms of PD-1 (E Ceccarello *et al*. manuscript in preparation) were engineered. These reversible modifications result in TCR-T cells with more antiviral-inflammatory but less direct cytotoxic effect or TCR-T cells that will not only lyse HCC cells but also modify the effects of PD-L1 expression in the TME. We hope that future clinical trials will evaluate the ability of T-cell therapy alone, or in combination with other strategies, to not only reduce HCC size through direct cytolysis but to also permanently modify the TME, and with this, improve our ongoing struggle against HCC.

## Data Availability

No new data were generated or analysed in support of this research.

## Abbreviations

AFP: alpha-fetoprotein
CAR: chimeric antigen receptor
HBV: Hepatitis B virus
HBV-HCC: HBV-related hepatocellular carcinoma
HBsAg: HBV surface antigen
HCC: Hepatocellular carcinoma
HCV: Hepatitis C virus
TAA: tumor-associated antigens
TCR: T-cell receptor
TMB: tumour mutational burden
TME: tumour microenvironment
